# Social and psychological capital in relation to social well-being among university students in Maragheh: A cross-sectional study

**DOI:** 10.1371/journal.pone.0353262

**Published:** 2026-07-16

**Authors:** Arman Latifi, Shahryar Mokhtary, Hossein Nemati, Ali Karimi Mirzanezam, Sara Rahimi

**Affiliations:** 1 Research Center for Evidence-Based Health Management, Maragheh University of Medical Sciences, Maragheh, Iran; 2 School of Management and Medical Informatics, Health Economics Department, Tabriz University of Medical Science, Tabriz, Iran; 3 Department of Nursing, Tabriz Islamic Azad University of Medical Sciences, Tabriz, Iran; 4 Student research committee, Maragheh University of Medical Sciences, Maragheh, Iran; 5 Department of Medical-Surgical Nursing, School of Nursing and Midwifery, Tabriz University of Medical Sciences, Tabriz, Iran; 6 Medicinal Plants Research Center, Maragheh University of Medical Sciences, Maragheh, Iran; Ajman University, UNITED ARAB EMIRATES

## Abstract

**Background:**

Social Capital and Psychological Capital are important determinants of individuals’ Social Well-Being. However, limited evidence exists on how these factors interact among university students in Iran. This study aimed to examine the relationships between Social Capital, Psychological Capital, and social well-being among Iranian university students.

**Methods:**

In this cross-sectional study, 460 students enrolled at universities in Maragheh County were selected using cluster sampling. Data were collected through self-reported questionnaires, including demographic information (age, gender, marital status, residence status, and parents’ education), the Psychological Capital Questionnaire, the Social Capital Questionnaire, and the social well-being Questionnaire, in accordance with ethical considerations. Data were analyzed using SPSS v24. Descriptive statistics summarized the sample characteristics. Pearson correlation coefficients were computed to assess relationships among variables. Multiple regression analysis was conducted to examine the predictive effects of Psychological Capital, Social well-being, and demographic factors (marital status, residence status, father’s education) on Social Capital, with a 95% confidence level.

**Results:**

Psychological Capital (mean ± SD: 99.11 ± 17.82) was positively associated with both Social Capital (70.02 ± 11.17) and social well-being (60.07 ± 6.12). Among demographic variables, marital status, residence status, and father’s education were significantly related to Social Capital. Multiple regression analysis showed that social well-being, Psychological Capital, and students’ residence status together explained a moderate proportion (22%) of the variance in Social Capital.

**Conclusions:**

The findings demonstrate significant positive relationships between Psychological Capital, social well-being, and Social Capital. Psychological Capital, social well-being, and students’ residence status together predicted 22% of the variance in Social Capital. These results highlight the importance of promoting Psychological and social well-being to enhance Social Capital among university students.

## Introduction

In recent years, Positive psychology has emphasized the presence of adaptive psychological strengths that contribute to students’ well-being and functioning. Contrary to traditional models that define mental health as the absence of illness, positive psychology emphasizes the presence of favorable traits such as hope, resilience, and self-efficacy—collectively referred to as psychological capital [1]. Psychological capital is a multidimensional construct comprising four core components: hope (goal-directed energy and planning), self-efficacy (confidence in task-specific abilities), resilience (capacity to bounce back from adversity), and optimism (positive expectations about future outcomes). Together, these dimensions are theorized to contribute to improved academic performance, life satisfaction, and interpersonal functioning [2,3]. In educational settings, higher levels of psychological capital have been linked with greater engagement, persistence, and emotional well-being among students [1,4]. Another construct gaining prominence in student well-being research is social well-being, defined by Keyes as individuals’ ability to function in society and maintain positive relationships within social networks [5]. Social Well-Being encompasses social integration, acceptance, contribution, actualization, and coherence, which collectively reflect the individual’s perceived connectedness and functionality within their community. Research has increasingly pointed to the critical role of social well-being in promoting psychological resilience, academic motivation, and life quality [6,7]. Central to both psychological and social functioning is the concept of social capital, which refers to the resources embedded in social relationships that facilitate collective action and individual advancement [8,9]. Social capital includes features such as trust, reciprocity, shared norms, and social participation. Students with greater access to social capital are more likely to benefit from emotional support, information exchange, and opportunities for collaboration—factors that are essential for thriving in university environments [10–12].

Recent research has suggested an association between psychological capital and social capital. Students with high levels of self-efficacy and resilience are more likely to build supportive social networks. Additionally, social well-being, as defined by Keyes, reflects individuals’ integration and functioning within society, and may contribute to social capital [13]. From a theoretical standpoint, this study integrates Luthans’ Psychological Capital Theory [1] with Bourdieu’s Theory of Social Capital [8] to propose a framework in which psychological capital and social well-being function as potential predictors of social capital.

Despite growing evidence that psychological capital and social well-being contribute to students’ academic and psychological well-being, the mechanisms through which these constructs shape social capital remain insufficiently understood. Social capital is recognized as a critical resource that enhances students’ academic engagement, mental health, and sense of belonging [9,14,15]. However, existing research has primarily examined psychological capital or social well-being in isolation, leaving unclear how these two constructs jointly predict students’ access to social networks, trust, and supportive relationships. Moreover, the overwhelming majority of studies have been conducted in Western contexts, where cultural norms surrounding social connectedness, community participation, and interpersonal trust differ significantly from those in Middle Eastern societies [16]. This lack of culturally diverse evidence limits the generalizability of prior findings and constrains theoretical development in non-Western settings. Given that cultural context shapes both psychological resources and social relational structures, there is a critical need for empirical studies that examine these relationships within underrepresented regions such as Iran. Therefore, the present study was undertaken to address this gap by investigating whether psychological capital and social well-being function as predictors of social capital among Iranian university students. By doing so, the study aims to advance cross-cultural understanding of student well-being and provide evidence that may inform context-sensitive interventions designed to strengthen students’ social integration and support systems.

Although previous studies have explored the role of psychological capital or social well-being in predicting student well-being, few have examined their joint relationship with social capital in higher education contexts. Moreover, existing studies are predominantly situated in Western or limited national settings, with insufficient attention to non-Western cultural contexts. This gap restricts the generalizability of findings and limits theoretical development. For example, Dong et al. demonstrated the mediating role of psychological capital in the association between interpersonal relationships and mental health among Chinese medical students [17], while Slåtten et al. examined the impact of psychological capital and social-contextual factors on study-related outcomes in Norway [18]. Similarly, Zhao et al. investigated the combined effects of psychological and social capital on mental health and happiness during COVID-19 in China [19]. However, despite such international evidence, research remains scarce in Middle Eastern settings. The present study addresses this gap by investigating the relationship between psychological capital, social well-being, and social capital among university students in Iran, thus providing novel evidence from a Middle Eastern context. Our findings contribute to the growing literature by clarifying the interplay between these variables and offering insights that may inform interventions to enhance student well-being and social connectedness.

## Materials and methods

### Study design and sampling

This cross-sectional study included students from three universities located in Maragheh County: Maragheh University of Medical Sciences, Islamic Azad University (Maragheh Branch), and Payame Noor University. Maragheh is a mid-sized urban city in north-western Iran’s East Azerbaijan province with roughly 175,000 inhabitants.

A multi-stage cluster sampling method was employed: each university was considered a cluster, faculties within each university were randomly selected, and students were then proportionally and randomly sampled from each faculty based on enrollment lists. This approach enhanced the representativeness of the sample and minimized selection bias. The minimum required sample size was initially estimated using Cochran’s formula for finite populations, assuming a 95% confidence level, 5% margin of error, and a 50% response distribution, which yielded approximately 384 participants. Given the use of cluster sampling, a design effect (DEFF) was applied; assuming a design effect of about 1.2, the adjusted sample size increased to roughly 460 individuals. This sample size also ensured sufficient statistical power for correlation and multiple regression analyses, in line with standard recommendations of at least 10–20 participants per predictor variable [20,21]. Using this approach allows for the detection of medium-sized effects while maintaining adequate representation across the three universities. A total of 498 eligible students were approached during the recruitment period. Of these, 460 agreed to participate and completed the questionnaires, yielding a response rate of 92.4%. Non-participation was mainly due to a lack of time or unwillingness to complete the full survey. Within each selected faculty, class rosters were obtained from administrative offices, and participants were randomly selected using computer-generated random numbers in proportion to faculty enrollment size. Data collection was conducted in classrooms and student common areas by trained research assistants, who explained the study objectives and obtained written informed consent before questionnaire administration.

#### Hypotheses.

Based on previous literature, we hypothesized that:

(1) Higher psychological capital and higher social well-being would be positively associated with higher levels of social capital;
(2) Psychological capital and social well-being would significantly predict social capital after adjusting for demographic variables;
(3) Specific demographic characteristics, including residence status and parental education, would show significant associations with students’ social capital.

#### Study objectives.

The primary objective of this study was to assess social capital as the main outcome variable. Psychological capital and social well-being were defined as the primary explanatory variables. Demographic characteristics including; marital status, place of residence, and parental education, were examined as additional predictors to explore their potential associations with social capital.

#### Variables.

In this study, social capital (total score of the Rafiei et al. questionnaire) was defined as the primary outcome variable. Psychological capital and social well-being (total scores of their respective validated instruments) were specified as the main explanatory variables. Demographic variables—including gender, marital status, residence status, father’s education, mother’s education, and household income—were treated as covariates and were evaluated for potential confounding effects. These variables were selected based on prior literature indicating their possible associations with social or psychological resources among university students.

#### Inclusion criteria.

All undergraduate and postgraduate students enrolled in any of the participating universities in Maragheh County were eligible to participate, regardless of nationality, ethnicity, gender, or field of study. Eligibility required willingness to participate, demonstrated through the provision of oral informed consent, as well as the ability to understand and respond to the study instruments in the language approved by the research team.

#### Exclusion criteria.

Individuals with a history of chronic illness, as specified in the study protocol, were excluded to minimize potential confounding effects on psychological or social functioning. Students who had experienced a major life event (e.g., bereavement of a close family member or parental divorce) within the past six months were also excluded due to the potential impact of acute stress on the primary study variables. Additionally, individuals with any condition that could interfere with accurate questionnaire completion or the ability to provide informed consent (e.g., unresolved language barriers or cognitive impairment) were excluded to maintain data integrity.

### Data collection method

Data were collected between 23 September 2023 and 20 February 2024. Data were collected using a four-part structured questionnaire comprising the following components: Demographic Questionnaire: Included items on age, gender, marital status, place of residence, parents’ educational level and occupation, and household income. Psychological Capital Questionnaire (PCQ): Developed by Luthans et al. (2007), (Copyright © 2007 by Fred Luthans, Bruce J. Avolio & James B. Avey. All rights reserved in all media. Published by Mind Garden, Inc., www.mindgarden.com. The use of this instrument was authorized through a research license issued to Arman Latifi by Mind Garden, Inc.), this 24-item scale consists of four subscales: hope, resilience, optimism, and self-efficacy (6 items each) [22]. Each item is rated on a 6-point Likert scale ranging from 1 (“strongly disagree”) to 6 (“strongly agree”), with higher scores indicating stronger psychological capital. The total score ranges from 24 to 144. The Persian version was validated by Bahadori Khosroshahi et al. (2012), reporting a Cronbach’s alpha of 0.85 [23]. Social Capital Questionnaire: Developed by Rafiey et al. (2015), this 20-item instrument measures five subscales: empathy and belonging (6 items), diverse interests (5 items), lifestyle diversity (4 items), trust (3 items), and social participation/cooperation (2 items). Responses are recorded on a 5-point Likert scale (1 = “strongly disagree” to 5 = “strongly agree”), yielding total scores ranging from 20 to 100. Reported internal consistency (Cronbach’s alpha) was 0.82 [24]. social well-being Questionnaire: Originally developed by Keyes (1998), this instrument includes 20 items across five dimensions: social actualization (4 items), social cohesion (3 items), social integration (3 items), social acceptance (5 items), and social contribution (5 items) (6). Each item is rated on a 5-point scale from 1 (“completely disagree”) to 5 (“completely agree”). Total scores range from 20 to 100, with higher scores reflecting better perceived social well-being. Reliability in Iranian samples was confirmed by Babapour (2009; α = 0.78) and Ahari (2012; α = 0.80) (20, 21). All the three instruments were selected based on their wide use in prior research, strong psychometric properties, and availability of validated Persian versions.

### Statistical analysis

Data were analyzed using SPSS version 24. Descriptive statistics (means, standard deviations, frequencies, and percentages) were calculated for all study variables. Pearson correlation coefficients were computed to examine associations among the main continuous variables and their subscales. Given the large number of pairwise correlations, the Bonferroni correction was applied to adjust the significance threshold and reduce the likelihood of Type I error; adjusted p-values are reported in the tables. The normality of continuous variables and regression residuals was evaluated using the Kolmogorov–Smirnov test, supplemented by visual inspection of histograms and Q–Q plots. Minor deviations from normality were deemed acceptable given the large sample size (n = 460), consistent with the Central Limit Theorem. Linearity was assessed through scatterplots between each predictor and the outcome variable. Homoscedasticity was examined using residual-versus-predicted value plots. Independence of errors was evaluated using the Durbin–Watson statistic. Multicollinearity was assessed through Variance Inflation Factor (VIF) and Tolerance values, all of which were within acceptable ranges. Prior to regression modeling, diagnostic analyses confirmed that model assumptions were adequately met. Multicollinearity was not problematic, with VIF values ranging from 1.08 to 1.76 and tolerance values ranging from 0.57 to 0.93. The Durbin–Watson statistic was 1.94, indicating independence of residuals. Visual inspection of residual histograms and Q–Q plots suggested approximate normality. Scatterplots of standardized residuals against predicted values indicated acceptable linearity and homoscedasticity without major outliers or influential observations. For comparisons of mean scores across demographic groups (as presented in Table 4), independent-samples t-tests were employed for binary variables (e.g., gender, residence status), and one-way ANOVA was used for variables with more than two categories (e.g., parental education, parental occupation). When ANOVA results were significant, post-hoc analyses with Bonferroni correction were applied. Multiple linear regression analysis was performed with social capital (total score) as the dependent variable. Based on theoretical frameworks and prior evidence, psychological capital and social well-being (total scores) were entered as the primary explanatory variables. Demographic variables (gender, marital status, residence status, and father’s education level) were included as potential confounders. Categorical predictors were dummy-coded, and the following reference categories were defined: male (gender), single (marital status), living with family (residence), and fathers with non-academic education (education level). A two-sided significance level of p < 0.05 was used for all analyses. To ensure adequate power for the regression analysis, an a priori power analysis was conducted using G*Power 3.1. For a model with six predictors, a medium anticipated effect size (f² = 0.15), α = 0.05, and power = 0.80 required a minimum of 98 participants. Additionally, a correlation-based estimate (anticipated effect size r = 0.15, α = 0.05, power = 0.80) suggested a required sample of at least 346 participants. The final sample of 460 students exceeded both thresholds, ensuring sufficient statistical power for the analyses.

**Table 4:** The difference of social capital, psychological capital, and social well-being according to demographic variables (n = 460).

Variables		Social Capital	P-value	Psychological Capital	P-value	social well-being	P-value
		Mean±SD		Mean±SD		Mean±SD	
Gender	Female	70.73 ± 11.30	0.057	99.64 ± 16.68	0.406	60.25 ± 5.82	0.384
	Male	68.64 ± 10.82		98.10 ± 19.86		59.73 ± 6.67	
Marital status	Married	65.32 ± 13.99	0.013	96.62 ± 21.03	0.369	58.94 ± 5.17	0.164
	Single	70.60 ± 10.65		99.42 ± 17.40		60.21 ± 6.22	
Residence status	University’s dormitory	72.59 ± 9.62	<0.001	100.57 ± 15.68	0.109	60.31 ± 5.32	0.425
	With family	67.96 ± 11.90		97.94 ± 19.32		59.89 ± 6.70	
Father’s educational level	Illiterate	69.55 ± 10.99	0.035	95.92 ± 15.29	0.662	61.64 ± 5.32	0.188
	Diploma	70.03 ± 11.02		99.31 ± 17.63		60.19 ± 6.15	
	Academic	75.25 ± 12.77		99.33 ± 18.92		59.40 ± 6.19	
Mother’s educational level	Illiterate	70.13 ± 13.14	0.814	95.79 ± 18.27	0.315	60.50 ± 4.45	0.884
	Diploma	69.82 ± 11.07		99.55 ± 17.89		59.99 ± 6.48	
	Academic	70.69 ± 10.03		99.76 ± 17.15		60.07 ± 6.12	
Father’s job	Unemployed	67.33 ± 9.25	0.588	89.05 ± 23.02	0.156	59.11 ± 7.03	0.052
	Worker	67.11 ± 11.01		96.66 ± 19.31		59.83 ± 5.72	
	Employee	69.97 ± 11.89		99.67 ± 18.46		59.82 ± 5.83	
	Free	70.24 ± 11.29		99.44 ± 17.33		60.07 ± 6.22	
	Retired	71.15 ± 9.10		100.71 ± 15.23		61.26 ± 6.21	
Mother’s job	Housewife	70.26 ± 11.45	0.130	99.32 ± 17.44	0.475	60.18 ± 6.26	0.212
	Employee	70.35 ± 9.87		99.78 ± 19.53		60.19 ± 4.84	
	Free	62.90 ± 11.66		93.18 ± 20.86		56.27 ± 8.70	
	Retired	66.60 ± 6.50		93.10 ± 15.73		59.40 ± 5.91	
Household income status	Income less than expenses	68.87 ± 9.93	0.198	98.39 ± 17.59	0.285	60.05 ± 7.17	0.492
	Income equals expenditure	70.44 ± 11.32		98.96 ± 17.65		60.16 ± 5.88	
	More income than expenses	66.29 ± 12.80		105.76 ± 22.06		58.35 ± 6.13	
Age		r = 0.031	0.502	r = 0.072	0.121	r = 0.027	0.558

### Ethics approval and consent to participate

This study was approved by the Research Ethics Committee of Maragheh University of Medical Sciences, Iran (Approval code: IR.MARAGHEHPHC.REC.1397.019). All procedures were conducted in accordance with relevant national research ethics guidelines and the principles of the Declaration of Helsinki (2013, Informed Consent) [25]. Participants were fully informed about the study’s objectives, procedures, confidentiality, and the voluntary nature of participation. All of the participants completed the written informed consent form.

## Results

In this study, among the 460 students, 303 (65.9%) were female and 157 (34.1%) were male. The average age of the participants was 20.90 years (standard deviation = 2.63), with ages ranging from 18 to 46 years the oldest participant was a non-traditional student enrolled part-time. (Table 1).

**Table 1:** Demographic characteristics of the participants (n = 460).

Variables		Frequency	Percentage (%)
Gender	Female	303	65.9
	Male	157	34.1
Marital status	Married	50	10.9
	Single	410	89.1
Residence status (place of residence)	University’s dormitory	205	44.6
	With family	255	55.4
Father’s education	Illiterate	28	6.1
	Diploma	314	68.3
	Academic	118	25.6
Mother’s education	Illiterate	58	12.6
	Diploma	327	71.1
	Academic	75	16.3
Father’s job	Unemployed	18	3.9
	Worker	18	3.9
	Employee	123	26.7
	Free	256	55.7
	Retired	45	9.8
Mother’s job	Housewife	366	79.5
	Employee	73	18.9
	Free	11	2.4
	Retired	10	2.2
Household income status	Income less than expenses	78	17.0
	Income equals expenditure	365	79.3
	More income than expenses	17	3.7
Age (year) ^*^ (18–46)		20.90 ± 2.63	

* Mean±SD.

The average and standard deviation of each component of social capital, psychological capital, and social well-being are presented in Table 2. According to this table, the average of social capital was measured at 70.02 ± 11.17, within a range of 34–97. The average of psychological capital was recorded with a mean and standard deviation of 99.11 ± 17.82, spanning from 33 to 144, while social well-being was measured at 60.07 ± 6.12, within a range of 35–81.

**Table 2:** Social capital, psychological capital, and social well-being in the study participants(n = 460).

Variables	Components	Observed Range*	Mean±SD
social capital	Empathy and belonging	9-30	22.38 ± 4.67
	Different interests	5-25	16.83 ± 4.92
	Different lifestyle	4-19	13.50 ± 3.25
	Trust	3-15	9.41 ± 2.14
	social participation and cooperation	2-10	7.87 ± 1.93
	Total	34-97	70.02 ± 11.17
psychological capital	Self-efficacy	7-36	25.07 ± 6.32
	Hope	7-36	25.54 ± 5.72
	Resilience	6-36	24.49 ± 5.13
	Optimism	6-36	23.99 ± 4.59
	Total	33-144	99.11 ± 17.82
social well-being	social actualization	4-17	11.85 ± 2.19
	Social integration	3-15	9.07 ± 2.31
	Social coherence	3-15	11.15 ± 2.69
	Social acceptance	7-24	15.13 ± 2.51
	Social contribution	5-22	12.87 ± 2.82
	Total	35-81	60.07 ± 6.12

* The theoretical ranges of each scale and subscale are provided in the Methods section. The ranges in this table correspond to observed scores in the sample.

The correlation coefficients between the components of social capital, psychological capital, and social well-being are presented in Table 3. As shown in Table 3, all components of psychological capital and social well-being exhibit a positive and significant relationship with social capital. Bonferroni correction was applied to control for multiple comparisons. Among all components, the strongest correlation within psychological capital was observed between hope and the empathy and belonging component of social capital (r = 0.423, p < 0.001). Similarly, within social well-being, the social coherence component showed the highest correlation with empathy and belonging in social capital (r = 0.479, p < 0.001). Additionally, the component of self-efficacy from psychological capital (r = 0.346) and the component of social coherence from social well-being (r = 0.302) have the highest correlation coefficients with the overall score of social capital (p < 0.001).

**Table 3:** Matrix of correlation coefficients between research variables.

	Empathy and belonging	Different interests	Different lifestyle	Trust	social participation and cooperation	Social Capital
Self-efficacy	0.400^*^ <0.001^**^	0.028 0.554	0.134 0.004	0.308 <0.001	0.391 <0.001	0.346 <0.001
Hope	0.423 <0.001	0.032 0.487	0.055 0.243	0.240 <0.001	0.350 <0.001	0.314 <0.001
Resilience	0.365 <0.001	0.058 0.212	0.090 0.054	0.181 <0.001	0.298 <0.001	0.291 <0.001
Optimism	0.318 <0.001	0.001 0.976	0.001 0.976	0.159 0.001	0.239 <0.001	0.205 <0.001
Psychological Capital	0.464 <0.001	0.037 0.429	0.091 0.049	0.279 <0.001	0.398 <0.001	0.360 <0.001
Social actualization	0.203 <0.001	0.087 0.061	0.106 0.022	0.178 <0.001	0.142 0.002	0.213 <0.001
Social integration	0.007 0.879	0.188 <0.001	0.052 0.269	0.110 0.018	0.050 0.287	0.083 0.076
Social coherence	0.479 <0.001	0.018 0.697	0.013 0.782	0.298 <0.001	0.322 <0.001	0.302 <0.001
Social acceptance	0.159 0.001	0.024 0.609	0.082 0.078	0.144 0.002	0.161 0.001	0.156 0.001
Social contribution	0.156 0.001	0.096 0.041	0.029 0.530	0.038 0.412	0.049 0.294	0.030 0.516
social well-being	0.273 <0.001	0.148 0.001	0.099 0.034	0.195 <0.001	0.254 <0.001	0.290 <0.001

* Pearson correlation coefficient.

** Indicates statistically significant correlation at p < 0.05 level.

The differences in the average scores of social capital, psychological capital, and social well-being based on demographic variables are presented in Table 4. marital status (p = 0.013), residency status (p < 0.001), and the father’s education level (p = 0.035) have a significant relationship with students’ social capital. Consequently, single students have higher average social capital scores compared to married students; students living in dormitories have higher scores than those living with their families; and students whose fathers hold university degrees score higher than those whose fathers have lower levels of education.

According to Table 4, social capital scores were significantly higher among single students compared to married ones, among dormitory residents compared to those living with family, and among students whose fathers had an academic education compared to those with less-educated fathers (p < 0.05).

Similarly, social well-being scores varied across some demographic subgroups, although these differences were generally less pronounced. No significant differences in psychological capital were observed across most demographic variables. The results emphasize that demographic characteristics such as marital status, place of residence, and parental education are associated with variations in students’ social capital and, to a lesser extent, their social well-being.

The results of the multiple linear regression analysis are presented in Table 5. This model indicates that after adjusting for potential confounding variables, psychological capital (B = 0.18, 95% CI: 0.13–0.23) and social well-being (B = 0.36, 95% CI: 0.21–0.52) are positively associated with social capital (p < 0.001), and, (*R*^*2*^ = 0.222). Beyond statistical significance, the observed effect sizes indicate meaningful practical relevance. The regression model explained 22.2% of the variance in social capital, which represents a moderate effect size according to conventional benchmarks in behavioral sciences. This suggests that psychological capital and social well-being are not merely statistically associated with social capital, but may also have meaningful implications for students’ real-world social functioning and connectedness.

**Table 5:** Multiple linear regression of the association between social capital, psychological capital, and social well-being.

Variables	B	SE	Standardized Coefficients Beta	95% CI	*p*-value
(Constant)	27.80	6.08		[15.83 to 39.76]	< 0.001^a^
Gender	1.91	0.98	0.081	[−0.008 to 3.84]	0.051
Marital status	−3.12	1.54	−0.08	[−6.15 to −0.09]	0.043
Residence status	3.69	0.96	0.16	[ 1.80 to 5.58]	< 0.001
Father’s education	−1.44	0.88	−0.06	[−3.18 to −0.30]	0.105
Psychological Capital	0.18	0.02	0.29	[ 0.13 to 0.23]	< 0.001
social well-being	0.36	0.07	0.20	[0.21 to 0.52]	< 0.001

R^2^ =0.222; Adjusted R^2^=0.212; F = 21.54; ^a^ statistically significant association.

### Discussion

The present study explored the relationship between psychological capital, social well-being, and social capital among university students. The results indicated that all components of psychological capital and social well-being were significantly and positively associated with social capital. These findings are consistent with previous research in Iran that has demonstrated similar associations between these constructs [26–28]. The findings support the theoretical assumption that internal psychological resources—such as hope, self-efficacy, resilience, and optimism—play a critical role in fostering students’ external social connections. Among the subcomponents, hope exhibited the strongest correlation with empathy and belonging, a key dimension of social capital. This result aligns with the studies of Khosroshahi (2012) and Azarabadi (2021), which emphasized the reciprocal influence of social support and hope [26,29]. When students feel emotionally supported and understood within their peer networks, they are more likely to maintain a positive outlook and engage more actively in social settings. Similarly, self-efficacy showed a strong positive relationship with overall social capital. Students who believe in their ability to succeed are more confident in initiating and maintaining social relationships, which reinforces their sense of social connectedness. This is in line with Bandura’s theory of self-efficacy [30], and consistent with prior findings in both Iranian and international contexts [17,29]. A potential mechanism underlying these associations is that higher Psychological Capital enables students to actively build and maintain supportive social networks, while greater social well-being enhances trust, reciprocity, and participation within social groups [31]. Cultural context may also shape these relationships; for example, in Iranian university settings, communal norms and family ties may reinforce social engagement, influencing the interplay between psychological resources and Social Capital.

Another important finding was that social coherence, a component of social well-being, had the strongest correlation with both empathy/belonging and overall social capital. This supports Hezarjaribi’s (2012) conclusion that social cohesion fosters resilience and trust within a community [32]. Individuals who perceive their social environment as predictable and meaningful tend to develop stronger interpersonal ties and coping mechanisms. The current findings are further reinforced by international studies. Recent international evidence further supports our findings. For example, Zhao et al. (2022) reported that psychological and social capital jointly contributed to better mental health and well-being among Chinese adults during the COVID-19 pandemic [19]. Similarly, Slåtten et al. (2023) found that psychological capital and supportive social environments significantly predicted academic outcomes among Norwegian university students [18]. More recently, Dong et al. (2025) demonstrated that psychological capital mediated the relationship between interpersonal relationships and mental health among Chinese medical students [17]. Together, these findings support the broader international relevance of the present results. Likewise, Selvaraj and Bhat (2018) demonstrated the predictive value of optimism and resilience for mental health among Indian and American students [33]. These findings, in combination with our own, highlight the cross-cultural relevance of psychological capital and social well-being as foundational resources for enhancing social capital, particularly in academic environments. Such evidence supports the broader applicability of interventions aimed at strengthening psychological strengths as a means of fostering social integration and support. The observed roles of optimism and resilience in this study are also conceptually supported. Optimism fosters trust and expectation of positive social outcomes, while resilience enables individuals to maintain social ties despite adversity [30,34]. These psychological strengths may facilitate students’ social integration and participation. It is important to note that the cross-sectional design limits causal inference; the observed associations do not imply temporal or causal relationships. Reliance on self-reported questionnaires may introduce response bias, and exclusion of individuals with chronic conditions or recent stressful events could underestimate variability in psychological capital and social well-being.

Demographic analysis revealed that marital status, residency (dormitory vs. living with family), and father’s education were significantly associated with social capital. Single students, dormitory residents, and those with academically educated fathers had higher social capital scores. These patterns may reflect greater opportunities for social interaction in communal settings. Similar trends were observed in previous studies conducted by Moradian et al. (2011), Rezaei et al. (2014), Irandoost et al. (2021), and Solhi et al. (2020) [34–38]. However, findings on marital status and parental education were not entirely consistent across studies, which may be attributed to cultural differences, research tools, or contextual variations [35,36,39,40]. No significant associations were found between social capital and age, gender, or income status in this study,results that partially align with or contrast prior findings [35–37,41]. While several Iranian studies have examined similar constructs, this study contributes by simultaneously analyzing psychological capital, social well-being, and Social Capital within a single model, specifically in Maragheh universities. From a theoretical perspective, Bourdieu’s Social Capital Theory helps interpret how individuals’ psychological capacities can shape resources within social networks, and Luthans’ Psychological Capital Theory explains why hope, resilience, and self-efficacy promote social engagement. These frameworks, combined with cultural considerations, provide insight into the observed relationships. The present study extends existing evidence in several important ways. First, although previous studies have examined associations between psychological resources and social outcomes, few studies, particularly in Iran, have simultaneously modeled psychological capital, social well-being, and social capital within a single multivariable framework. This allowed us to estimate the independent contribution of each construct while adjusting for demographic covariates. Second, our findings suggest that psychological capital may be a stronger predictor of social capital than social well-being, as reflected by the larger standardized regression coefficient. This indicates that internal psychological resources such as hope, resilience, and self-efficacy may play a particularly influential role in facilitating trust, participation, and social connectedness among university students. Third, this study contributes evidence from a Middle Eastern cultural context, where collectivist norms, stronger family bonds, and residential arrangements (e.g., dormitory versus family living) may shape the development of social capital differently from Western settings. Together, these findings provide incremental theoretical and contextual evidence for understanding the mechanisms linking psychological and social resources in higher education.

From a theoretical perspective, the present findings reinforce both Bourdieu’s and Luthans’ frameworks. Bourdieu conceptualized social capital as resources embedded within social networks that facilitate social mobility and access to opportunities. Our findings suggest that students with stronger psychological resources may be better positioned to develop and utilize these network-based resources. Likewise, Luthans’ Psychological Capital Theory proposes that hope, resilience, self-efficacy, and optimism function as positive psychological assets that enhance adaptive functioning. The current results support this proposition by showing that these internal resources are positively associated with stronger social connectedness and social capital.

## Conclusion

The present cross-sectional study identified significant associations between psychological capital, social well-being, and social capital among Iranian university students. Students who reported higher levels of psychological resources and social well-being also tended to report more favorable levels of social capital. Nonetheless, due to the correlational nature of the research design, these findings should be interpreted cautiously. The study does not permit conclusions regarding causality, the temporal ordering of variables, or the possibility of reciprocal relationships among them. Future longitudinal and experimental research is needed to determine whether psychological capital and social well-being contribute to the development of social capital, whether social capital enhances these constructs, or whether the relationships operate bidirectionally. Despite these methodological constraints, the present findings provide valuable preliminary evidence that contributes to the understanding of social and psychological factors relevant to students’ well-being in the Iranian higher-education context.

### Strengths of the study

This study possesses several methodological strengths. First, it was conducted on a relatively large and diverse sample of university students from multiple institutions, which enhances the representativeness of the findings. Second, the use of validated and culturally adapted Persian instruments for assessing psychological capital, social well-being, and social capital supports the reliability and cultural appropriateness of the measures. Third, the application of multivariate statistical analyses enables a comprehensive examination of the relationships among variables. While certain limitations remain,such as the cross-sectional design, reliance on self-reported data, and cluster sampling without adjustment for design effect,these methodological strengths contribute to the overall rigor and interpretability of the study, providing valuable insights into the interplay between psychological resources, social well-being, and social capital among university students.

### Limitation

One key limitation of this study is its geographical scope, as data were collected exclusively from universities located in Maragheh County. Therefore, caution is warranted when generalizing the findings to broader student populations within Iran or to other countries, where sociocultural, economic, and institutional contexts may differ in meaningful ways that affect psychological capital, social well-being, and social capital. Although one participating institution was a medical university, the sample also included students from non-medical universities, which improves the broader relevance of the findings. Therefore, the observed relationships may be partially generalizable to students in other academic disciplines and regions of Iran. However, regional sociocultural differences, institutional environments, and student demographics may influence these associations; thus, generalization should be made cautiously. Moreover, the cross-sectional design inherently restricts the interpretation of the results to associations rather than causal relationships. This design does not allow determination of the temporal ordering among psychological capital, social well-being, and social capital; therefore, no conclusions can be drawn regarding whether these variables influence one another over time or whether reciprocal or reverse causal pathways exist. Although statistically significant associations were identified, future longitudinal or experimental studies are required to clarify the directionality and potential mechanisms underlying these relationships. The reliance on self-report questionnaires represents an additional limitation, as responses may be subject to social desirability, recall bias, or measurement error. Furthermore, students with chronic illnesses or recent major stressful life events were excluded, which may limit the representativeness of the sample and reduce generalizability to the wider student population. While several demographic variables were included in the regression model, other potential confounders or effect modifiers may not have been fully accounted for. Finally, although cluster sampling was used, no adjustment was applied for design effect, which may have resulted in underestimation of standard errors and inflated statistical significance. Despite the appropriateness of Pearson correlations and multiple linear regression for the research objectives, future studies may benefit from employing more advanced analytical approaches, such as mediation analysis or structural equation modeling (SEM),particularly within longitudinal or quasi-experimental frameworks. Such designs would provide deeper insight into the direct and indirect pathways linking psychological capital and social well-being to social capital.

### Suggestions

The present findings may have practical implications for university counseling services and student support programs. Counseling centers may consider implementing interventions aimed at strengthening psychological capital, such as resilience training, self-efficacy enhancement, and hope-based counseling. Universities may also promote peer-support initiatives, group mentoring, and community-building programs to improve students’ social well-being and facilitate stronger social capital. In light of the associative nature of the findings, future research is encouraged to employ longitudinal, multi-wave, or intervention-based designs to clarify the directionality and underlying mechanisms of the observed relationships. Additionally, qualitative or mixed-method approaches may provide deeper insights into how students perceive and construct Social Capital within their educational and cultural contexts. Further studies should also examine the influence of demographic factors, such as marital status, residential status, and parental education, across diverse universities and sociocultural settings. Moreover, researchers could consider employing structural equation modeling (SEM) to explore potential mediation, moderation, and interaction effects among psychological capital, social well-being, and social capital. Comparative analyses across fields of study (e.g., medical, humanities, engineering) and public versus private universities could further illuminate contextual differences. Finally, while the current findings suggest potential areas of interest for higher-education policymakers, any practical or programmatic initiatives aimed at enhancing students’ psychological or social well-being should be informed by evidence from studies capable of establishing causal relationships.

## Supporting information

S1 File Data. (XLSX)
